# Supplementary material for: Recreating the Trabecular Outflow Tissue on Implantable, Micropatterned, Ultrathin, Porous Polycaprolactone Scaffolds
Source: Bioengineering (Basel). 2023 Jun 2;10(6):679. doi: 10.3390/bioengineering10060679 (PMC10294786; doi:10.3390/bioengineering10060679)
Supplement: Supplementary file 1 [file bioengineering-10-00679-s001.zip › bioengineering-1883970-supplementary.pdf]

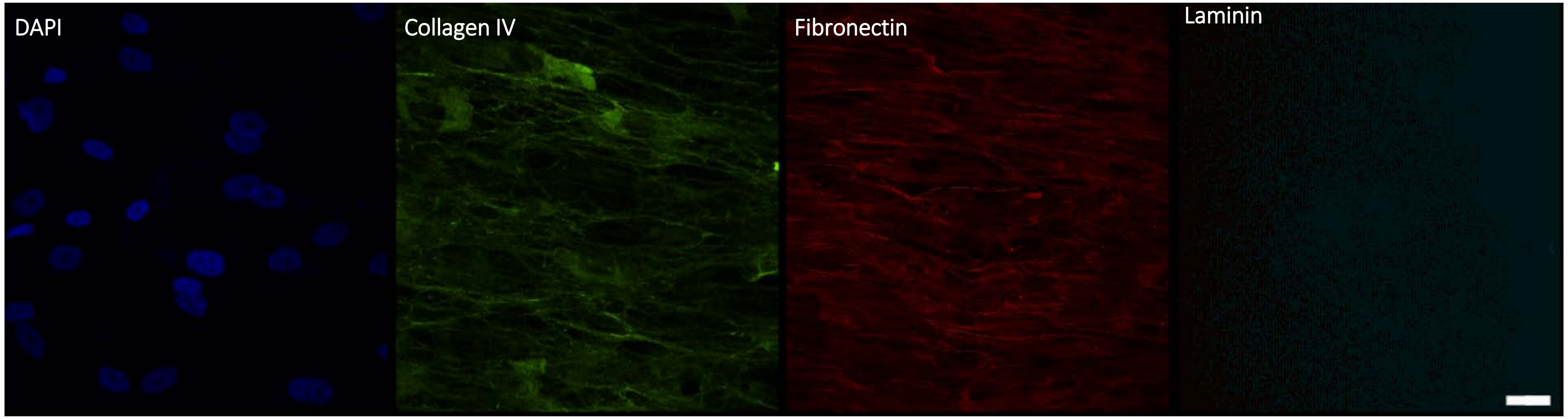


**Figure S1 .** Expression of ECM proteins, collagen, fibronectin and laminin of HTM cells grown on gelatin-coated coverslips.

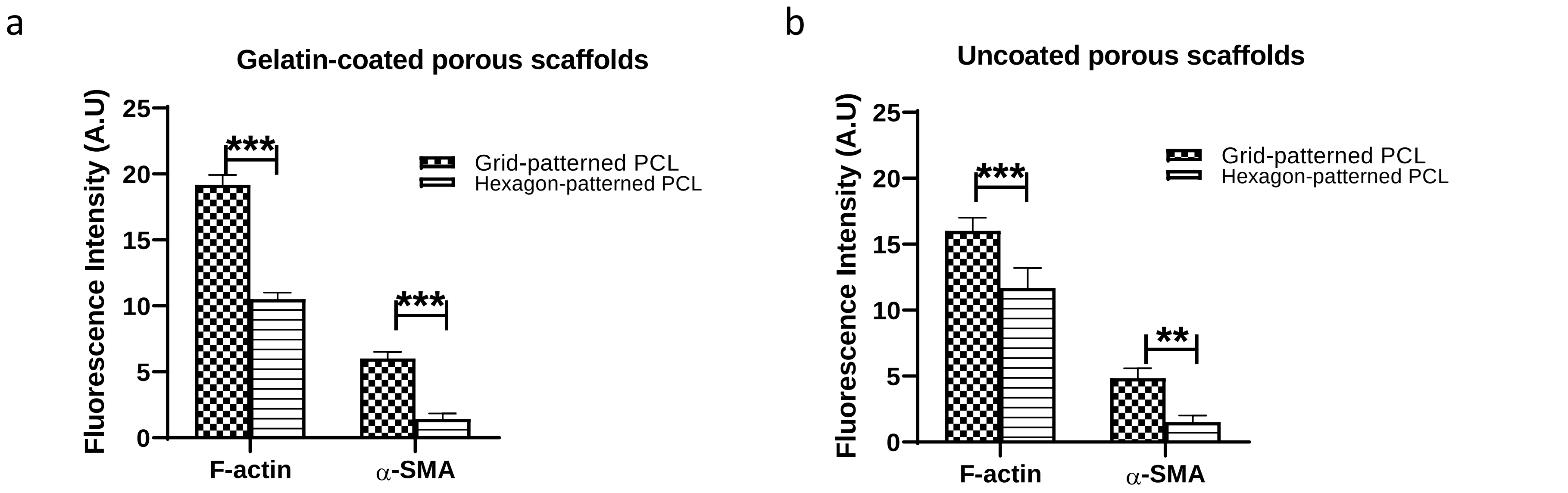


**Figure S2.** ImageJ quantification of fluorescence intensity of F-actin and α-SMA expression in HTM cells grown in grid- and hexagon-patterned PCL scaffolds with (a) and without (b) gelatin-coating. *, $p < 0.05$. **, $p < 0.01$. ***, $p < 0.001$.
